# Applying Endoscopic Interventions to Inflammatory Bowel Disease-Related Strictures

**DOI:** 10.1093/crocol/otaa066

**Published:** 2020-10-30

**Authors:** Amy L Lightner

**Affiliations:** Department of Colorectal Surgery, Cleveland Clinic, Cleveland, Ohio, USA

Crohn’s disease is an idiopathic chronic inflammatory condition of the gastrointestinal tract that can exhibit 3 phenotypes including inflammatory, penetrating, of fibrostenotic disease.^1^ When chronic inflammation results in fibrosis, there can be irreversible bowel wall damage that requires surgical intervention. Unfortunately, up to one-third of Crohn’s patients develop a stricture.^2^ Despite the advent of monoclonal antibody therapy, rates of surgery for Crohn’s disease have not significantly decreased with up to 60%–80% requiring major abdominal surgery.

Surgery is not curative; rather, it palliates symptoms of Crohn’s disease and its associated bowel wall damage. Surgery is also not without complication. While surgery can often be performed in a minimally invasive fashion with a bowel sparing approach, postoperative morbidity including intra-abdominal sepsis and disease recurrence can occur in up to 30% and 80%, respectively.^3–6^ In addition, up to a third of Crohn’s patients who undergo an ileocecal resection will develop a postoperative stricture at the anastomotic site.^7^ Thus, finding minimally invasive endoscopic approaches to manage intestinal Crohn’s disease can help prevent operative morbidity and potentially short bowel by avoid surgery or spacing out repeated surgical interventions.

Previously described endoscopic approaches include endoscopic balloon dilation (EBD), endoscopic needle knife stricturotomy, insulated-tip knife sticturotomy, stent placement, and local injection with long-acting corticosteroids.^8–12^ Currently, EBD is the primary treatment approach for endoscopic treatment of both inflammatory bowel disease (IBD) and non-IBD related strictures.^13–18^ However, more than half of Crohn’s patients who undergo EBD require multiple EBD interventions and at least one-third require surgery shortly after dilation as salvage therapy.^19^

More recently, endoscopic stricturotomy is being increasingly utilized for inflammatory bowel disease-related strictures secondary to inflammatory primary strictures and postoperative anastomotic strictures.^10,20^ Needle knife stricturotomy in IBD was first reported in 2011 by Dr. Shen who performed the technique in ileal pouch strictures no amenable to EBD.^12^ In that series of 406 therapeutic endoscopies, there were only 2 perforations, and ad a median follow up of 9.6 years, 87% were able to retain their pouches. In a later study of 85 IBD patients, endoscopic needle knife therapy was effective in the treatment primary and secondary IBD related strictures with only 15% requiring subsequent surgery. In addition, there were only 10 adverse events in the series of which one required surgery.^10^ Interestingly, in another series of endoscopic stricturotomy vs ileocecal resection for anastomotic strictures at ileocecal anastomoses, need for subsequent surgery was 10% and 11%, respectively with decreased morbidity in the stricturotomy group.^21^ In addition, another recent study looked at endoscopic stricturotomy in IBD and non-IBD related strictures and, again IBD patients tended to have a higher tendency for maintaining surgical remission after stricturotomy with only 12% requiring subsequent surgical intervention.^22^

In this issue of *Crohn’s and Colitis 360*, Nabeeha Mohy-ud-din and Gursimran Kochhar reported on endoscopic stricturotomy in 11 IBD patients treated with 12 procedures in Pittsburg between 2018 and 2020 with a median follow up of xx. Four of the patients had ulcerative colitis with an ileal pouch, 2 had Crohn’s colitis, and 5 had a diagnosis of Crohn’s of the small bowel or colon. Nearly all (91%) had undergone bowel-related surgery prior to the endoscopic stricturotomy, and 73% were on monoclonal antibody therapy for disease control. The primary outcome was technical success of the procedure as defined by traversing the stricture with a colonoscopy after the procedure. Secondary outcomes were defined as symptom improvement, need for additional procedures, need for hospitalization, and complications including bleeding, infection or perforation related to the procedure. Consistent with previous studies, largely out of one single institution, the authors again found technical success and safety with 92% achieving successful treatment and one patient (9%) experiencing bleeding from the procedure. Of the 11 procedures, 4 required both EBD and stricturotomy to fully open the stricture for traversion with the scope.

While endoscopic procedures clearly have a role in the treatment of IBD-related strictures, there are limitations to highlight which underscore the importance of both patient selection and physician experience with various techniques. The commonly used endoscopic procedure, EBD, while effective results in high rates of symptomatic recurrence and need for repeat procedures or surgical intervention; a meta-analysis of 2664 EBD procedures found that at 5 years following EBD, 80% had required repeat dilation and 75% had required surgical intervention.^23^ In addition, the majority of studies report that one-third of patients who undergo endoscopic dilation of primary or anastomotic strictures require surgical intervention within 2 to 5 years.^24–27^ These results question the utility of EBD as a bowel conserving strategy. While stricturotomy appears superior to EBD with regard to the need for subsequent intervention, several of the studies have not had long-term follow-up limiting the conclusiveness of bowel-sparing effectiveness with avoidance of surgery.^20^ For example, the study of stricturotomy vs ileocecal resection only followed patients for a mean of 0.8 years (0.2–1.7),^21^ and another study of 85 IBD patients undergoing stricturotomy were followed for a median of 0.9 years (0.3–1.8).^10^ Thus, while these results paint a good picture for procedural safety and short-term prevention of surgery, it remains unclear as to whether surgical rates are decreased in the long run, a true marker of success with the procedure.

In addition, rate of re-intervention in all these endoscopic studies remains high. Even in the study highlighted in this journal, one-third of patients needed intervention with both EBD dan stricturotomy. In addition, in one study of IBD and non-IBD patients, stricture improvement was only seen in 20% of IBD patients on post-procedure endoscopy with 50% of patients requiring repeated sessions of stricturotomy, EBD, and both combined.^22^ Another similar study reported 61% requiring repeated EBD, stricturotomy, or both following endoscopic edoscopic stricturotomy.^10^ Thus, the need for repeated interventions in not trivial and should be taken into account vs undergoing more definitive surgical intervention. The median follow up of the article herein was only 144 days limiting the long term data and need for both repeated endoscopic intervention as well as surgical intervention.

In addition, while endoscopic intervention may work well for short strictures as highlighted in the aforementioned articles, there are many patients with long-segment primary and anastomotic strictures, for which endoscopic intervention may be more risky and/or not feasible. The median length of the series herein was 10.25 ± 4.36 mm. This is similar to that reported in the previous series, all of which typically conclude that endoscopic intervention should only be used in strictures less than 4 cm.

Lastly, it is important to consider malignancy can be found within strictures, especially colonic strictures in long-standing inflammation of Crohn’s colitis and ulcerative colitis in which 2%–6% have a malignancy.^8,9,28,29^ Therefore, strictures, especially those that cannot be adequately biopsied or prevent adequate surveillance of proximal bowel should be surgically resected in order to obtain adequate tissue histology.

In conclusion, Nabeeha Mohy-ud-din and Gursimran Kochhar have added a nice additional patient series to the literature regarding endoscopic stricturotomy, which has largely been dominated by one previous institution. While a small series than some others reported, they confirmed the safety and feasibility, along with short term efficacy of this minimally invasive technique. While not applicable to all patients and all strictures, this potentially surgery sparing technique should be considered in appropriately selected patients.

## Notes

Disclosures: A.L. received consultant fees from Takeda and has no financial disclosures.

Data Availability Statement: Data sharing is not applicable to this article as no new data were created or analyzed.
